# Supplementary material for: Drivers of systemic male-female allele frequency divergence in humans
Source: bioRxiv. 2026 Jan 6:2026.01.06.698007. Preprint. [Version 1] doi: 10.64898/2026.01.06.698007 (PMC12803062; doi:10.64898/2026.01.06.698007)
Supplement: Supplement 1 [file media-1.pdf]

Supplemental Information

Drivers of systemic male-female allele frequency divergence in humans

Matthew Ming and Arbel Harpak

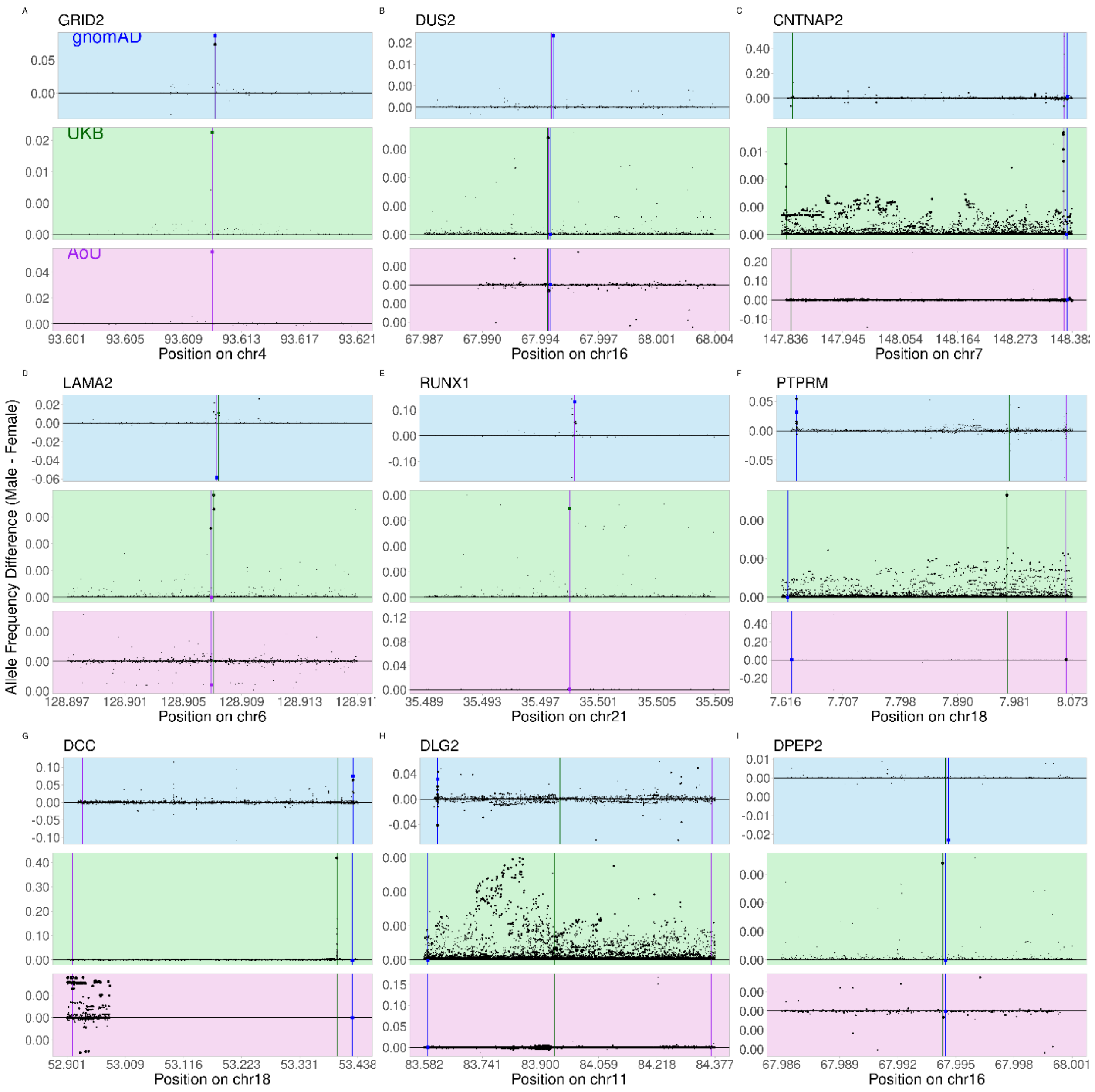

Supplemental Figure 1: Allele frequency differences in cross-study significant genes that were not significant by MAGMA. We identified nine genes that do not show MAGMA significance but do show cross-study significance by Fisher's exact test. As in Fig. 1, each point is a single SNP, with its size reflecting Chi-squared test p-value. The y-axis shows male-minus-female allele frequency, polarized by the allele with higher frequency in males in UKB. Plots are colored by the dataset they show (blue for gnomAD, green for UKB, purple for AoU). Colored square points represent the lowest p-value site in each dataset.

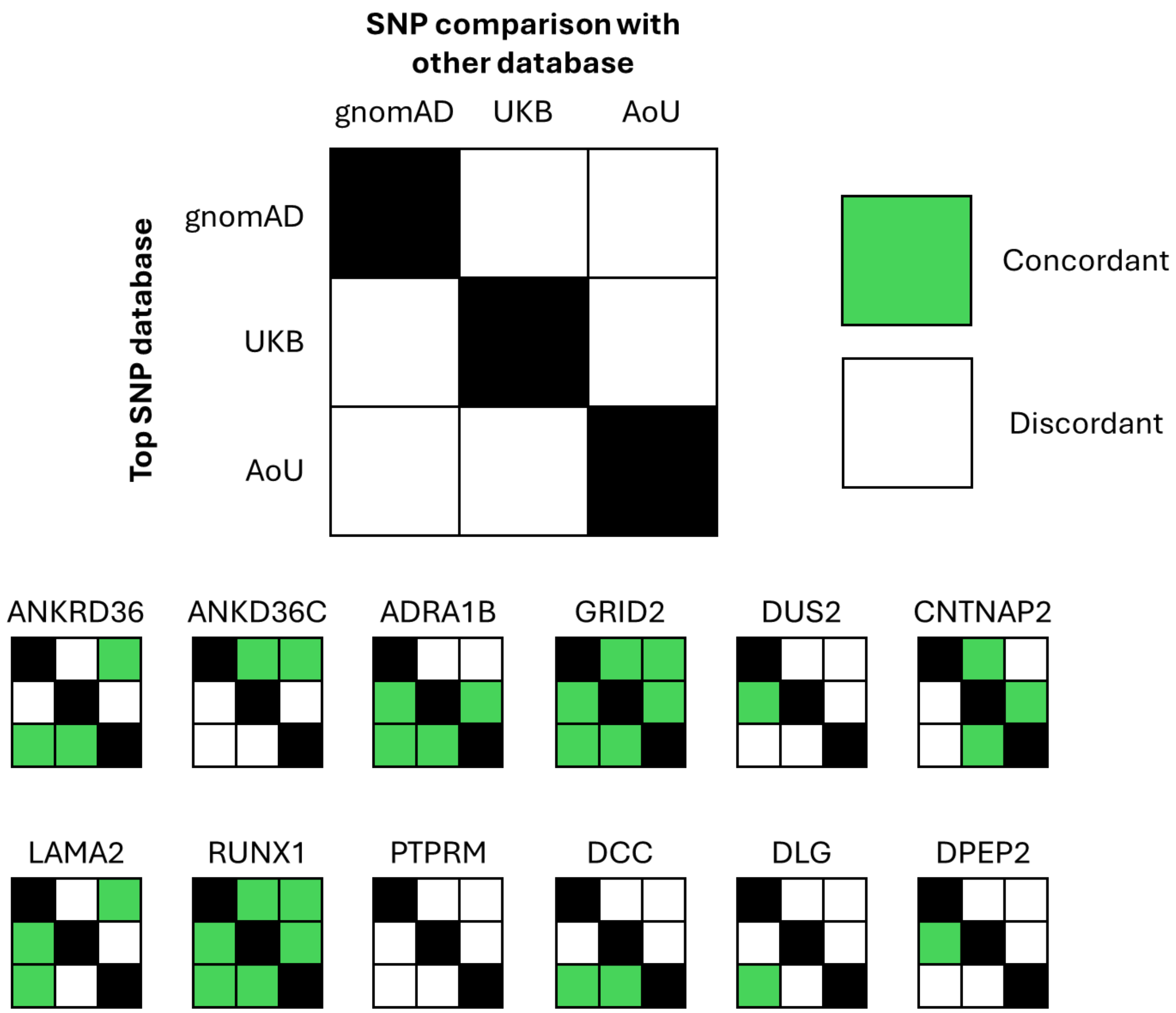

Supplemental Figure 2: Cross-study sign concordance of lead SNPs. We report the pairwise sign concordance for the most significant SNP in each study. The study from which a given lead SNP was obtained is in each row, and the study with which the sign is compared is in each column. Cells are colored green if the sign is concordant, and white if the sign is discordant.

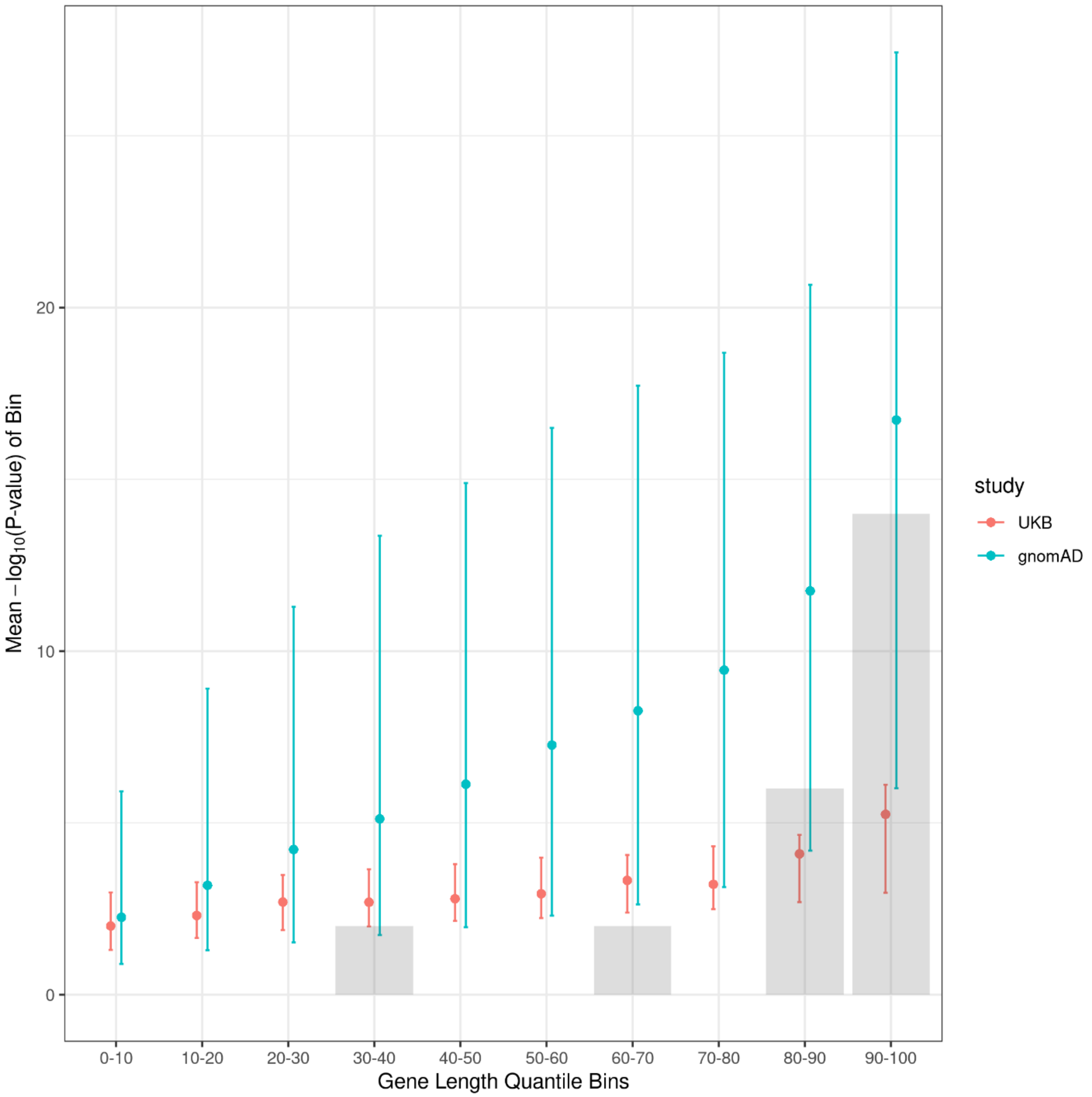

Supplemental Figure 3: Gene-level p-value tends to decrease as gene length increases. Cross-study significant genes are enriched for long gene lengths. The x-axis is gene size binned into ten equally sized bins by quantile. Teal and red points represent the means in gnomAD and UKB respectively, while the blue and red lines represent the 5th to 95th confidence intervals for those datasets. The grey boxes represent relative abundances of the cross-study significant genes in each quantile bin.

GENE	P-value (gnomAD)	P-value (UKB)	P-value (AoU)
ANKRD36C	8.8537e-05	1.956e-06	8.7594e-08
ANKRD36	0.054531	1.5296e-06	0.00048075
GRID2	0.95061	0.71392	0.003257
ADRA1B	0.0022114	0.00033647	1.2207e-05
LAMA2	0.97	0.8497	0.80159
CNTNAP2	0.77751	0.76475	0.56456
DLG2	0.41545	0.63698	0.54942
DPEP2			0.055514
DUS2	0.80445	0.33035	0.060242
PTPRM	0.31745	0.47281	0.92597
DCC	0.70643	0.11567	0.030954
RUNX1	0.99765	0.74864	0.76731
**COLOR KEY**	$p < 0.001$	$p < 0.01$	$p < 0.05$

Supplemental Table 1: Gene-wide aggregated p-values calculated using MAGMA. P-values represent the probability of a summary statistic calculated by summing p-values calculated for all SNPs within a gene, weighted by their correlation with each other. Cells are colored by their significance, with darkest being most significant ($p < 0.001$) and lightest being least ($p < 0.05$).

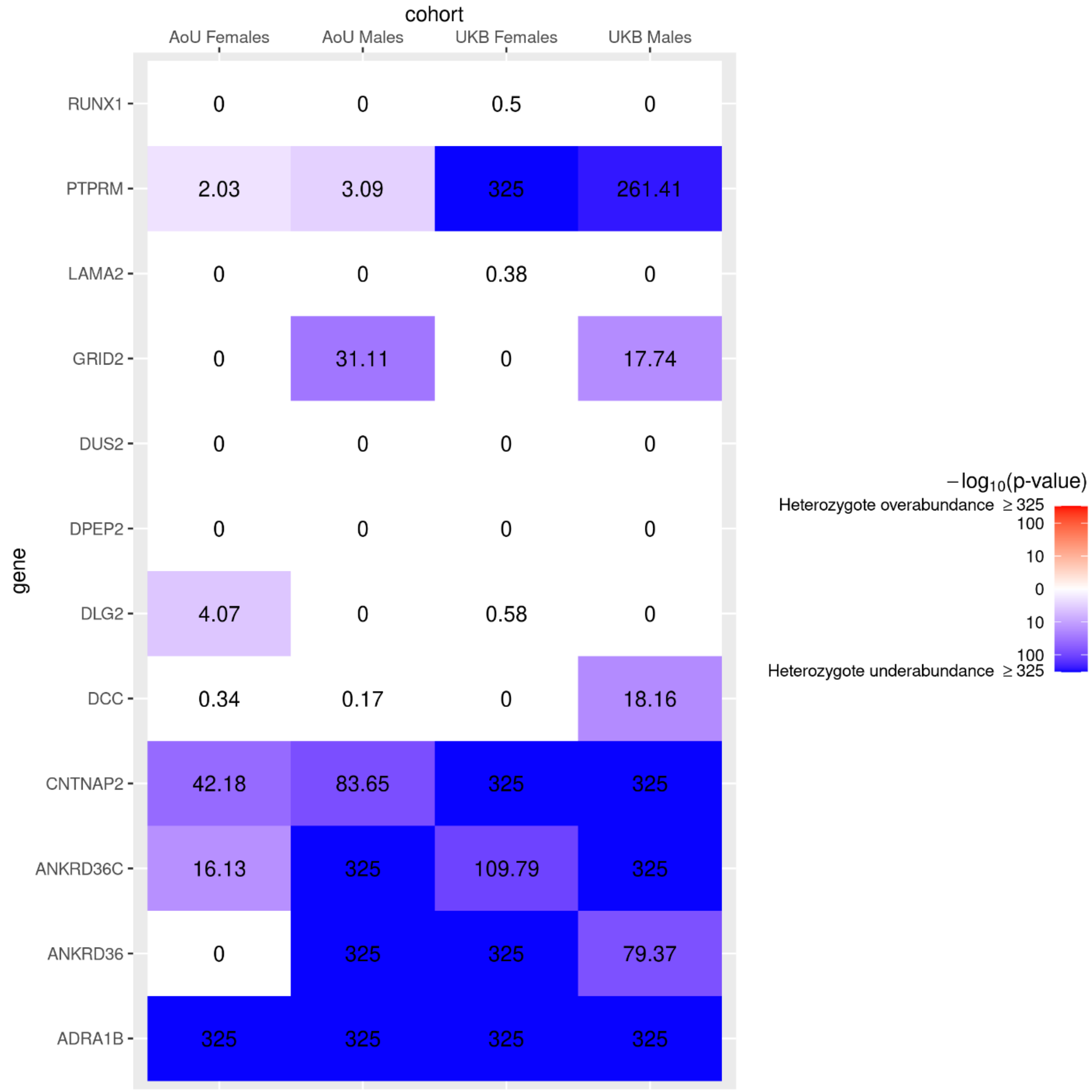

Supplemental Figure 4: Comparison of deviations from Hardy-Weinberg equilibrium for most significant SNP in cross-study significant genes. Shown are -$\log_{10}$(p-values) for deviations from Hardy-Weinberg Equilibrium (HWE). Each row corresponds to a cross-study significant gene, and each column corresponds to one sex in either UKB or AoU. The intensity of colors represents p-value for deviation from HWE by exact test, with darker colors being lower p-values; red indicates an overabundance of heterozygotes compared to expected under HWE, and blue indicates an underabundance of heterozygotes; the color intensity is scaled logarithmically to highlight the full range of values.

Gene Name	Shared across bridges
GRID2	Not shared across bridges
ANKRD36C	Not shared across bridges
DCC	Not shared across bridges
ANKRD36	Shared across bridges
DUS2	No data
PTPRM	Non-significant results

Supplemental Table 2: Genes putatively under SDS which are related to spermatogenesis and fertility are also among those that are not shared across cytoplasmic bridges. We find through comparison with results from Bhutani et al. (2021) that several of the cross-study significant genes associated with spermatogenesis and fertility are also not shared across cytoplasmic bridges during spermatogenesis, indicating that these could contribute directly to individual sperm phenotype from sperm haplotype.
